# Prevalence of vitamin D deficiency in patients with spinal cord injury at admission: a single-centred study in the UK

**DOI:** 10.1017/jns.2023.12

**Published:** 2023-02-20

**Authors:** Samford Wong, Honglin Dong, Shashivadan P. Hirani, Irina Gainullina, Ibrahim Ussef, Allison Graham

**Affiliations:** 1 National Spinal Injuries Centre, Stoke Mandeville Hospital, Aylesbury, UK; 2 Centre for Health Services Research, Department of Health Service and Research Management, School of Health and Psychological Sciences, City, University of London, London, UK; 3 The Royal Buckinghamshire Hospital, Aylesbury, UK

**Keywords:** 25(OH)D, Body mass index, Creatinine, Hyponatraemia, Spinal cord injury, Vitamin D deficiency

## Abstract

Vitamin D deficiency is prevalent in patients with chronic spinal cord injury (SCI) and has been implicated as an aetiologic factor of osteoporosis and various skeletal and extra-skeletal issues in SCI patients. Few data were available regarding vitamin D status in patients with acute SCI or immediately assessed at hospital admission. This retrospective cross-sectional study evaluated vitamin D status in SCI patients at admission to a UK SCI centre in January–December 2017. A total of 196 eligible patients with serum 25(OH)D concentration records at admission were recruited. The results found that 24 % were vitamin D deficient (serum 25(OH)D < 25 nmol/l), 57 % of the patients had serum 25(OH)D < 50 nmol/l. The male patients, patients admitted in the winter–spring time (December–May), and patients with serum sodium < 135 mmol/l or with non-traumatic causes had a significant higher prevalence of vitamin D deficiency than their counterparts (28 % males *v.* 11⋅8 % females, *P* = 0⋅02; 30⋅2 % in winter–spring *v.* 12⋅9 % in summer–autumn, *P* = 0⋅007; 32⋅1 % non-traumatic *v.* 17⋅6 % traumatic SCI, *P* = 0⋅03; 38⋅9 % low serum sodium *v.* 18⋅8 % normal serum sodium, *P* = 0⋅010). There was a significant inverse association of serum 25(OH)D concentration with body mass index (BMI) (*r* = −0⋅311, *P* = 0⋅002), serum total cholesterol (*r* = −0⋅168, *P* = 0⋅04) and creatinine concentrations (*r* = −0⋅162, *P* = 0⋅02) that were also significant predictors of serum 25(OH)D concentration. Strategies for systematic screening and efficacy of vitamin D supplementation in SCI patients need to be implemented and further investigated to prevent the vitamin D deficiency-related chronic complications.

## Introduction

Vitamin D, a fat-soluble pre-hormone, is a unique essential nutrient with limited natural food sources mostly of animal origin such as oily fish, red meat and egg yolk, and is mainly produced in the skin exposed to ultraviolet B (UVB) radiation from the sun^(1)^. Apart from its classical role in regulation of calcium and phosphate homeostasis as well as its skeletal effect, vitamin D also plays important roles in the modulation of cell growth, neuromuscular and immune function and anti-inflammation^(2)^. Although 1,25-dihydroxyvitamin D (active form of vitamin D) plays an active role in metabolism, its half-life is less than 4 h, while the half-life of 25-hydroxyvitamin D (25(OH)D, a metabolite of vitamin D) is around 2–3 weeks, thus serum 25(OH)D levels have been used to assess vitamin D status^(2)^. However, different authorities use different threshold of serum 25(OH)D to define vitamin D deficiency, for example, 25 nmol/l for Scientific Advisory Committee on Nutrition^(2)^ in the UK, 30 nmol/l for the Institute of Medicine, and 50 nmol/l for the European Food Safety Authority and Endocrine Society and no current consensus exists^(3)^. Vitamin D deficiency is prevalent worldwide reported as 9⋅9 % in the USA^(4)^, 7⋅4 % in Canada^(5)^, 4⋅6–30⋅7 % in Western Europe^(6)^ (defined as serum 25(OH)D < 30 nmol/l) and 18⋅8 % during winter and spring and 7⋅5 % during summer and autumn in the UK (defined as serum 25(OH)D concentration < 25 nmol/l)^(7)^. Evidence from observational studies shows that vitamin D deficiency is significantly associated with increased risks to musculoskeletal disease such as osteomalacia and non-musculoskeletal health outcomes including hypertension, obesity, cardiovascular disease (CVD) and diabetes, mortality from respiratory diseases and reduced lung functions, immune responses^(8,9)^ and advanced cancers (metastatic or fatal)^(10)^, although some large-scaled randomised controlled trials (RCTs) could not confirm the effect of vitamin D supplementation on the lower risk of fractures among generally healthy midlife and older adults^(11)^ or of the major adverse cardiovascular events in postmenopausal women^(12)^ and middle-aged and older adults^(13)^.

Previous studies have highlighted the prevalence of vitamin D deficiency in chronic spinal cord injury (SCI) patients^(14,15)^, and it is recommended that a 25(OH)D level be checked in individuals with chronic SCI^(16)^. Vitamin D deficiency has been implicated as an aetiologic factor responsible for osteoporosis and various skeletal and extra-skeletal issues in SCI patients^(17,18)^. This could be due to decreased mobility, prolonged institutionalisation and reduced exposure to sunlight following SCI^(19,20)^. However, there are very few reports regarding vitamin D status in patients with acute SCI or immediate assessment of vitamin D status at admission. Therefore, it is imperative to investigate whether patients are previously vitamin D deficient before hospital admission. The aim of the study was to evaluate vitamin D status indicated by serum 25-hydroxyvitamin D (25(OH)D) concentrations in SCI patients admitted to a UK SCI centre in 2017 and to assess the characteristics of vitamin D deficiency in this patient group.

## Method

This was a one-year, retrospective, point-prevalence study. The data were collected from a UK SCI centre during January to December 2017. Formal ethical permission to conduct the study was not required by the institution's review board as it did not involve active patient participation.

A 30-item cross-sectional questionnaire was distributed to the clinicians at the SCI centre (IG, IU and AG). The questionnaire consisted of three sections: the first section collected individual's baseline demographics (at the time of data collection), level and cause of SCI, weight and body mass index (BMI) and presence of co-morbidities; the second section collected routine blood biochemistry and haematology data including 25(OH)D within 7 d of admission; the third section recorded if the patient is on vitamin D, testosterone replacement therapy, anabolic steroid, antibiotic or diuretics.

The questionnaire (refer to the Supplementary material) was approved by the local clinical audit department. Participating centre was reassured that all data would be treated anonymously.

The inclusion criteria were SCI patients with serum 25(OH)D records. Patients without serum 25(OH)D record within 7 d of hospital admission were excluded from the study. There were 421 patients in total who were admitted to the SCI hospital centre in 2017, among which only 197 patients had serum 25(OH)D records. One patient was removed from data analysis as an outlier due to extremely high serum 25(OH)D concentration (226 nmol/l). Therefore, a total of 196 SCI patients were included in the study and data analysis. The categorical data (variables) included in the study are shown in Table 1. Instead of using four seasons, two seasons covering the whole year was adopted, defined as summer–autumn (June–November) and winter–spring (December–May) used in other studies^(7,21)^. Though different thresholds of serum 25(OH)D were used to define vitamin D deficiency, most studies with SCI patients used serum 25(OH)D < 50 nmol/l as a threshold of vitamin D deficiency, and serum 25(OH)D > 75 nmol/l as optimal or sufficient vitamin D status^(14)^. The present study defined vitamin D as serum 25(OH)D < 25 nmol/l recommended by SACN^(2)^ since the study was conducted in the UK, but also reported the percentage of patients with serum 25(OH)D < 50 nmol/l, 50–75 nmol/l and >75 nmol/l. The body weight categories were defined as underweight (BMI <18⋅5 kg/m^2^), normal weight (BMI 18⋅5–24⋅9 kg/m^2^) and overweight and obese (BMI ≥25 kg/m^2^)^(22)^. American Spinal Injury Association Impairment Scale (AIS) was categorised as complete (no motor or sensory function is preserved in the sacral segments S4–S5) and incomplete (the rest of SCI)^(23)^. The spinal nutrition screening tool (SNST) score was categorised as normal (≤10) and malnourished (>11)^(24)^. The serum sodium categories were defined as low sodium or hyponatraemia (sodium <135 mmol/l), normal sodium or normonatraemia (135–145 mmol/l), and high sodium or hypernatraemia (>145 mmol/l)^(25)^. The continuous data (variables) collected are shown in Table 2, including age, onset of SCI (days), BMI, SNST score, blood total cholesterol, high-density lipoprotein (HDL), cholesterol/HDL ratio, albumin, C-reactive protein (CRP), potassium, urea, creatinine, haematocrit, haemoglobin, white cell count, sodium and 25(HOH)D concentration.

**Table 1.** Number of records for categorical variables in SCI patients

Categorical variables	*N* (%)	Categorical variables	*N* (%)
Gender	194	AIS category	183
Female	51 (26 %)	Complete	76 (42 %)
Male	143 (74 %)	Non-complete	107 (58 %)
Age	196	Level of SCI	186
<65 y	144 (73 %)	Cervical	88 (47 %)
≥65 y	52 (27 %)	Non-cervical	98 (53 %)
Ethnicity	79	Onset SCI	178
White	73 (92 %)	≤180 d	92 (52 %)
Non-white	6 (8 %)	>180 d	86 (48 %)
BMI	101	Pressure ulcer	119
Normal weight	48 (47⋅5 %)	Yes	16 (13 %)
Underweight	5 (5 %)	No	103 (87 %)
Overweight/obesity^a^	48 (47⋅5 %)	Grade 3 or above ulcer	100
Seasons	196	Yes	10 (10 %)
Summer–autumn	70 (36 %)	No	90 (90 %)
Winter–spring	126 (64 %)	Serum sodium	192
SNST	98	≤135 mmol/l	36 (19 %)
Normal	77 (79 %)	136–145 mmol/l	154 (80 %)
Malnourished	21 (21 %)	>146 mmol/l	2 (1 %)
Cause of SCI	180		
Traumatic	102 (57 %)		
Non-traumatic	78 (43 %)		

*N*, number; SCI, spinal cord injury; AIS, American Spinal Injury Association impairment scale; BMI, body mass index; SNST, spinal nutrition screening tool.

a One patient's BMI was removed from data analysis as an outlier (60 kg/m^2^).

**Table 2.** Continuous variables of SCI patients

Continuous variables	*N*	Mean	sd
Serum 25(OH)D (nmol/l)	196	50⋅1	28⋅0
Age (years)	196	50⋅5	18⋅6
Length of hospital stay (days)	173	100⋅0	105⋅1
Onset of SCI (days)	178	2923⋅6	4883⋅9
BMI (kg/m^2^)	101	25⋅7	5⋅9
SNST score	98	8⋅9	2⋅8
Total cholesterol (nmol/l)	155	4⋅7	1⋅2
HDL (nmol/l)	156	1⋅0	0⋅3
Cholesterol/HDL ratio	156	5⋅1	2⋅0
Albumin (g/l)	190	34⋅3	6⋅4
CRP (nmol/l)	189	24⋅4	42⋅5
Potassium (mmol/l)	189	4⋅2	0⋅4
Urea (mmol/l)	192	4⋅8	1⋅8
Creatinine (μmol/l)	192	54⋅6	18⋅3
Haematocrit (%)	194	40⋅0	10⋅0
Haemoglobin (g/l)	194	126⋅9	20⋅9
White Cell Count (×10^9^/l)	194	10⋅1	14⋅0
Sodium (mmol/l)	192	138⋅2	4⋅0

*N*, number; SCI, spinal cord injury; BMI, body mass index; SNST, spinal nutrition screening tool; HDL, high-density lipoprotein; CRP, C-reactive protein.

Categorical variables (Table 1) were presented as frequency and percentage (%), while continuous variables were presented as mean ± standard deviation (sd). Statistical analysis was performed by Statistical Package for Social Sciences (SPSS) version 16.0 (SPSS Inc., Chicago, IL, USA). Normality of data distribution was tested by Kolmogorov–Smirnov. Vitamin D status (percentage of vitamin D deficiency) was compared between different groups using Pearson *χ*^2^. Mann–Whitney *U* test and Kruskal–Wallis were used to compare serum 25(OH)D between different groups. Correlation and single linear regression were performed to analyse the relationship of serum 25(OH)D with other continuous variables. The statistically significant level was set up as *P* ≤ 0⋅05 two-tailed. *Post hoc* power calculation was performed using G* Power (version 3.1.9.7) (Heinrich-Heine-Universität, Düsseldorf, Germany) to compute achieved power of 99⋅1–99⋅9 % (*χ*^2^ tests – Goodness-of-fit tests: Contingency tables; Analysis: *Post hoc*: Compute achieved power). The calculations were based on proportions of vitamin D deficiency in subgroups (genders, seasons, low and normal sodium, traumatic and non-traumatic groups).

## Results

### Descriptive characteristics of the SCI patients

Table 1 shows the categorical variables of the 196 SCI patients with valid 25(OH)D data. Most of the patients were male (74 % *v*. female 26 %), and younger adults (73 % for <65 y *v*. 27 % for ≥65 y). Seventy patients (36 %) were admitted to hospital during the summer–autumn period (June–November) *v*. 126 patients (64 %) during the winter–spring period (December–May). Only 101 patients had BMI records, among which 5 were underweight, 48 were normal weight and 48 were overweight/obese. The majority of patients with ethnicity records were white (92 % *v*. non-white 8 %). Among 180 patients with records, 57 % were traumatic *v*. 43 % non-traumatic SCI, and 42 % were complete *v*. 58 % incomplete SCI.

Table 2 shows the continuous variables collected from SCI patients. The average age of the patients was 50⋅5 (sd 18⋅6) years old (18–90 y). The average BMI was 25⋅7 (sd 5⋅9) kg/m^2^ (16–46 kg/m^2^). The mean of serum 25(OH)D concentration was 50⋅1 (sd 28⋅0) nmol/l (20⋅0–159⋅6 nmol/l).

### Vitamin D status of the SCI patients

Around a quarter (24 %) were vitamin D deficient (serum 25(OHO)D < 25 nmol/l), 57 % of patients had serum 25(OHO)D < 50 nmol/l, only 16 % of patients had serum 25(OH)D > 75 nmol/l that was recommended as optimal level^(26)^.

### Comparisons of serum 25(OH)D between groups

Table 3 shows male patients had a significant lower average serum 25(OH)D than females (47⋅6 nmol/l *v*. 58 nmol/l, *P* = 0⋅02). Serum 25(OH)D concentration was significant lower in the winter–spring period (December–May) than that in the summer–autumn period (June–November) (48⋅2 nmol/l *v*. 53⋅7 nmol/l, *P* = 0⋅04). There was no significant difference in serum 25(OH)D in other categorical groups.

**Table 3.** Serum 25(OH)D concentrations in different categories

Categorical variables	*N*	Mean (nmol/l)	sd	*P*-value*	Categorical variables	*N*	Mean (nmol/l)	sd	*P*-value*
Gender	194				AIS category	180			
Female	51	58⋅0	30⋅5	0⋅02	Complete	76	49⋅6	30⋅5	0⋅42
Male	143	47⋅6	26⋅8		Non-complete	107	50⋅7	26⋅6	
Age	196				Level of SCI	186			
<65 y	144	49⋅8	28⋅0	0⋅79	Cervical	88	47⋅7	28⋅7	0⋅18
≥65 y	52	51⋅1	28⋅4		Non-cervical	98	51⋅4	27⋅5	
Ethnicity	79				Onset SCI	178			
White	73	48⋅9	26⋅6	0⋅68	Acute ≤180 d	92	48⋅1	26⋅3	0⋅47
Non-White	6	63⋅4	54⋅2		Chronic >180 d	86	52⋅0	29⋅5	
BMI	101				Pressure ulcer	119			
Normal weight	48	52⋅2	30⋅7	0⋅09	Yes	16	53⋅8	29⋅7	0⋅66
Underweight	5	71⋅8	34⋅3		No	103	50⋅4	27⋅9	
Overweight/obesity	48	42⋅6	26⋅0		Grade 3 or above ulcer	100			
Seasons	196				Yes	10	57⋅7	31⋅2	0⋅42
Summer–autumn	70	53⋅7	25⋅2	0⋅04	No	90	49⋅9	28⋅4	
Winter–spring	126	48⋅2	29⋅4		Serum sodium	192			
SNST	98				≤135 mmol/l	36	45⋅7	29⋅4	0⋅09
Normal	77	47⋅0	29⋅2	0⋅40	136–145 mmol/l	154	51⋅8	27⋅4	
Malnourished	21	50⋅7	27⋅6		>146 mmol/l^a^	2	54⋅5	42⋅3	
Cause of SCI	180								
Traumatic	102	53⋅2	29⋅4	0⋅09					
Non-traumatic	78	46⋅3	26⋅5						

*N*, number; AIS, American Spinal Injury Association impairment scale; SCI, spinal cord injury; BMI, body mass index; SNST, spinal nutrition screening tool.

a Sodium category of >146 mmol/l was excluded in the analysis due to small sample size (*n* 2).

* Mann–Whitney *U* test.

### Comparisons of the percentage of vitamin D deficiency between groups

**Fig. 1.**
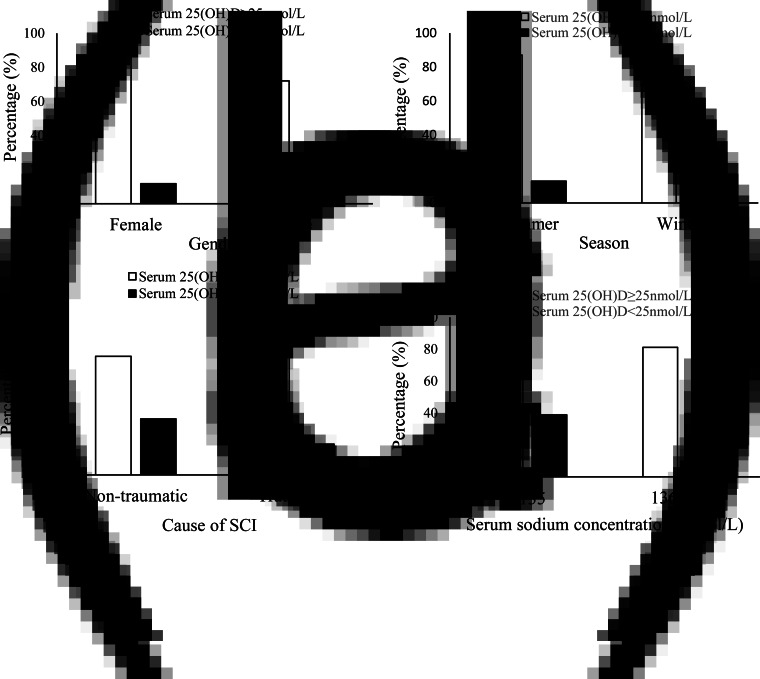
Percentage of patients with a serum 25(OH)D < 25 nmol/l. (a) Between gender (*P* = 0⋅02); (b) between seasons (*P* = 0⋅007); (c) between cause of SCI (*P* = 0⋅03); (d) between low and normal serum sodium concentrations (*P* = 0⋅01). SCI, spinal cord injury; Na, sodium. Pearson *χ*^2^ was used.

When serum 25(OH)D was categorised into serum 25(OH)D < 25 nmol/l (vitamin D deficiency) and serum 25(OH)D ≥ 25 nmol/l, a higher prevalence of vitamin D deficiency was observed in males (28 % males *v*. 11⋅8 % females, *P* = 0⋅02, Fig. 1(a)), in patients admitted to hospital during the winter–spring period (30⋅2 % in winter–spring *v*. 12⋅9 % in summer–autumn, *P* = 0⋅007, Fig. 1(b)), in patients with non-traumatic causes (32⋅1 % non-traumatic *v*. 17⋅6 % traumatic, *P* = 0⋅03, Fig. 1(c)), and in patients with low serum sodium (Na) concentrations or hyponatraemia (38⋅9 % low Na *v*. 18⋅8 % normal Na, *P* = 0⋅01, Fig. 1(d)). There was no significant difference in percentage of patients with serum 25(OH)D < 25 nmol/l between other categorical variables (data not shown).

### Correlation of serum 25(OH)D with other continuous variables

There was a significant inverse correlation of serum 25(OH)D with BMI (*r* = −0⋅311, *P* = 0⋅002), total serum cholesterol (*r* = −0⋅168, *P* = 0⋅04) and serum creatinine (*r* = −0⋅162, *P* = 0⋅02) (Table 4). The single linear regression (Table 5) shows that BMI, total cholesterol and creatinine were significant predictors of serum 25(OH)D concentration, which accounted for 8⋅8, 2⋅2 and 2⋅1 % of 25(OH)D variance, respectively. An increase of 1 BMI (kg/m^2^) was associated with a reduction in serum 25(OH)D by approximately 1⋅6 nmol/l, while an increase of 1 nmol/l total cholesterol was associated with a reduction in serum 25(OH)D by 4⋅1 nmol/l. Similarly, an increase of 1 μmol/l serum creatinine was associated with a reduction of serum 25(OH)D by 0⋅25 nmol/l.

**Table 4.** Correlation of serum 25(OH)D with other continuous variables

	*r*	*P*-value*	*N*
Age	−0⋅005	0⋅94	196
Onset of SCI (days)	−0⋅018	0⋅81	178
BMI	−0⋅311	0⋅002	101
SNST score	0⋅047	0⋅65	98
Total cholesterol	−0⋅168	0⋅04	155
HDL	0⋅048	0⋅55	156
Cholesterol/HDL ratio	−0⋅106	0⋅19	156
Albumin	0⋅043	0⋅56	190
CRP	0⋅054	0⋅46	189
Potassium	−0⋅082	0⋅26	189
Urea	0⋅021	0⋅77	192
Creatinine	−0⋅162	0⋅02	192
Haematocrit	−0⋅015	0⋅84	194
Haemoglobin	−0⋅055	0⋅45	194
White Cell Count	−0⋅003	0⋅97	194
Sodium	0⋅074	0⋅31	192

*r*, correlation coefficient; *N*, number.

* Pearson's correlation.

**Table 5.** Simple linear regression analysis summary for serum 25(OH)D concentration (dependent variable)

Independent variables (predictors)	Adjusted *R*^2^	*P-*value (ANOVA)	Constant	Unstandardised beta (B)	Standardised beta (β)	95 % CI for B
BMI	0⋅087	0⋅002	88⋅5	−1⋅552	−0⋅311	(−2⋅50, −0⋅605)
Total cholesterol	0⋅022	0⋅04	67⋅8	−4⋅093	−0⋅168	(−7⋅926, −0⋅059)
Creatinine	0⋅021	0⋅02	64⋅3	−0⋅250	−0⋅162	(−0⋅467, −0⋅033)

BMI, body mass index; CI, confidence interval.

## Discussion

The present study investigated the vitamin D status in SCI patients when admitted to a UK SCI centre and found that 24 % were vitamin D deficiency (serum 25(OH)D < 25 nmol/l). Male patients, patients admitted in winter time, and patients with hyponatraemia or caused by non-traumatic conditions had worse vitamin D status. In addition, BMI, blood total cholesterol and creatinine showed inverse association serum 25(OH)D and were significant predictors of serum 25(OH)D in this population.

The present study showed 24 % vitamin D deficient (serum 25(OH)D < 25 nmol/l) in SCI patients, with 30⋅2 % in winter–spring *v*. 12⋅9 % in summer–autumn (*P* = 0⋅007), much higher than the general population reported in a large cohort of 440 581 UK Biobank participants^(7)^ which showed 18⋅6 % of vitamin D deficiency in winter–spring and 7⋅5 % in summer–autumn period. Our results also showed male SCI patients had a significant higher prevalence of vitamin D deficiency compared with female SCI patients (28 % *v*. 11⋅8 %, *P* = 0⋅02), while Sutherland's results showed similar vitamin D deficiency between genders (7⋅4 % for males *v.* 7⋅6 % for females in the summer–autumn period, and 19⋅2 % for males *v.* 18⋅5 for females in the winter–spring period)^(7)^. Obviously, male SCI patients had a much higher prevalence of vitamin D deficiency than the general public.

Traumatic SCIs are most commonly caused by falls, road traffic accidents, sport injury and violence, whereas non-traumatic SCIs are normally caused by degenerative, inflammatory, neoplastic and infectious conditions^(27)^. Our results showed that a significant higher prevalence of vitamin D deficiency was observed in non-traumatic than traumatic SCI patients (32⋅1 % *v*. 17⋅6 %, *P* = 0⋅03), which is also higher than the general population at 18⋅6 % in winter–spring and 7⋅5 % in summer–autumn period^(7)^.

Hyponatraemia is defined as a serum sodium concentration <135 mmol/l^(25)^. Animal studies have shown that chronic hyponatraemia significantly reduced bone mineral density by approximately 30 % compared with normonatraemic control rats^(28)^. Observational studies in adults^(28)^ and elderly^(29)^ also found that hyponatraemia is significantly associated with increased odds of osteoporosis at the hip (odds ratio (OR) 2⋅85; 95 % confidence interval (CI) 1⋅03–7⋅86; *P* < 0⋅01) and with an increased risk of vertebral fractures and incident non-vertebral fractures. The classical role of vitamin D in bone health is well acknowledged and vitamin D deficiency has been implicated as an aetiologic factor responsible for osteoporosis and various skeletal and extra-skeletal issues^(30)^. Both hyponatraemia and vitamin D deficiency have been associated with gait disturbances, falls and fractures^(30)^. An Italian retrospective study in 5097 outpatients who were referred to health check in 2013 showed that hyponatraemic participants had significantly lower levels of serum vitamin D than normonatraemic patients (55 nmol/l *v*. 60 nmol/l, *P* = 0⋅02), along with a significantly higher rate of vitamin D deficiency defined as serum 25(OH)D < 50 nmol/l (41⋅8 % *v*. 36⋅1 %, *P* = 0⋅03)^(30)^, which was supported by the result of the present study that found a significant higher vitamin D deficiency rate in hyponatraemic patients than that in normonatraemic patients (38⋅9 % hyponatraemia *v*. 18⋅8 % normonatraemia, *P* = 0⋅010). It is still unknown of the relationship of serum 25(OH)D and sodium^(30)^. Nevertheless, this result indicates that serum sodium should be tested in vitamin D deficiency or vice versa in SCI patients.

The present study found a significant inverse association of serum 25(OH)D concentration with BMI, which aligns with substantial evidence from observational studies^(31)^. Volumetric dilution is the most accepted explanation, while vitamin D, being fat soluble, can also be stored in cutaneous and visceral adipose tissues, resulting in lower 25(OH)D levels in overweight and obese individuals^(32)^. Regarding vitamin D deficiency is a cause or an outcome of obesity, it may be a complex of mutual influence because vitamin D receptors are expressed on adipose cells and have a role in the function of those cells^(33)^.

Similarly, our results showed an inverse association of serum 25(OH)D with serum total cholesterol concentrations, which was supported by the evidence from observational studies^(34)^. A systematic review and meta-analysis^(35)^ including 41 randomised trials with a total of 3434 participants showed that vitamin D supplementation reduced the levels of serum total cholesterol, low-density lipoprotein cholesterol and triglycerides but not HDL-cholesterol, indicating a causal relation of vitamin D deficiency with dyslipidaemia, a major risk factor of CVD^(36)^. Research has shown that SCI was associated with a significant increased odds of heart disease (adjusted OR 2⋅72, 95 % CI 1⋅94–3⋅82) and stroke (adjusted OR 3⋅72, 95 % CI 2⋅22–6⋅23)^(37)^, which highlighted the need for vitamin D deficiency being identified and rectified to reduce risk factors for CVD in SCI patients.

The present study also found a significant inverse association of serum creatinine with serum 25(OH)D concentration. Creatinine level tends to represent the renal function. All SCI patients with serum creatinine records were within the normal range of serum creatinine concentrations apart from two (112 and 162 μmol/l). Though limited data are available in this area, a cross-sectional study found a significant inverse correlation between serum 25(OH)D and creatinine concentrations in 60 Saudi Arabian patients with the end-stage renal disease^(38)^. The mechanism of the possible protective role that vitamin D may play in kidney disease, is thought to be due to vitamin D's suppression of the renin-angiotensin-aldosterone system (RAAS) leading to improved glomerular filtration rate^(39)^. However, some evidence showed a significant increase in serum creatinine concentrations after administration of vitamin D receptor activator, paricalcitol 2 μg a day for 7 consecutive days without changing glomerular filtration rate^(40)^ or found that a 10 % increase in serum 25(OH)D levels causes a 0⋅3 % decrease in estimated glomerular filtration rate (eGFR) in a Mendelian randomisation study^(41)^. The above conflicting results are worthy of further research regarding vitamin D, blood creatinine and kidney function.

## Limitations of the study

We only analysed data from one SCI centre, and multicentre involvement will be more representative of vitamin D status in the SCI patients. We had serum 25(OH)D data at admission, without follow-up data available about the vitamin D treatment and health outcomes. Some patients had a long onset period before admission, which made our results inapplicable to acute SCI patients at admission. Due to the incomplete data collection, when multivariate regression was applied, very few samples (*n* 55) were included in the analysis, therefore the single simple regression was used instead. In addition, among 421 SCI patients admitted, less than half (*n* 197) had serum 25(OH)D records, indicating that more attention is needed to make vitamin D status screened and treated in SCI patients at an earlier stage. In addition, we did not collect data about if patient already taking vitamins supplement prior SCI / on transfer to SCI centre.

In conclusion, the prevalence of vitamin D deficiency in SCI patients at admission to hospital is higher than the general population, particularly in some subgroups such as male, patients admitted during the winter–spring time, patients with low blood sodium concentrations and with non-traumatic causes. Strategies for systematic screening and efficacy of vitamin D supplementation in SCI patients at the admission stage need to be implemented. We expect our results will raise the awareness that higher prevalence of vitamin D deficiency not only exists in chronic SCI patients but also in SCI patients when admitted to hospital, and vitamin D status test should become routine for SCI patients at hospital admission. Patients with vitamin D deficiency should be treated and should follow the guideline of the National Institute for Health and Care Excellence (NICE) regarding ‘Management of vitamin D deficiency or insufficiency’^(42)^. Further study of the efficacy of vitamin D supplementation on prevention of chronic complications of SCI patients including bone mineral density, body composition and CVD is warranted.

## Supplementary material

For supplementary material accompanying this paper visit https://doi.org/10.1017/jns.2023.12. click here to view supplementary material
