# Supplementary material for: Prevalence of vitamin D deficiency in patients with spinal cord injury at admission: a single-centred study in the UK
Source: J Nutr Sci. 2023 Feb 20;12:e24. doi: 10.1017/jns.2023.12 (PMC9947757; doi:10.1017/jns.2023.12)
Supplement: Supplementary file 1 [file S2048679023000125sup001.pdf]

Date of audit: ____________

Date of SCI ____________

Age [ ] Yrs

Sticker

Cause of SCI: ____________

Gender [ ]

SCI Level [ ]

Cause of SCI: ____________

ASIA [ ]

Social History

Cigarettes: ____________

ETOH: ____________

Anthropometry

Weight ______ Kg

BMI [ ] Kg/m2

Use of vitamin D supplement? Yes / No

If yes: __________________ Name, dose and frequency

Use of testosterone replacement therapy? Yes / No

If yes: __________________ Name, dose and frequency

Use of anabolic sterioid? Yes / No

If yes: __________________ Name, dose and frequency

Pressure ulcer? Yes / No

Grade 3 or above pressure ulcers? Yes / No

Trachestomy Yes / No

On antibiotics ? Yes / No

Indication of antibiotics? ______________________________

Fluid balance [ ]

Fluid intake [ ]

Output [ ]

From fluid balance chart

On other course of antibiotics in last 30 days Yes / No

Diruetics? Yes / No **If Yes, which one:**________________

Name, dose and frequency

Blood pressure (mm/Hg): ________________

Blood Biochemistry +/- 7 days of admission

Fasting / Random BMs		Sodim		CRP	
Total Cholesterol		Potassium			
LDL		Urea			
HDL		Creatinine			
TG		*Heamatocrit*			
HBA1C		Hammoglobin			
Albumin		Whie cell counts			
CRP		Iron			
vitamin D		Testestrone			
Total chol / HDL ratio					
